# Realistic dielectric response of high temperature sintered ZnO ceramic: a microscopic and spectroscopic approach†

**DOI:** 10.1039/d0ra04273k

**Published:** 2020-08-18

**Authors:** Sidra Ibadat, Muhammad Younas, Shaista Shahzada, Muhammad Nadeem, Tahir Ali, Muhammad Javed Akhtar, Simone Pollastri, Ubaid-Ur Rehman, Ibraheem Yousef, Rao Tahir Ali Khan

**Affiliations:** Department of Physics, Faculty of Basic and Applied Sciences, International Islamic University Islamabad Pakistan; Physics Division, PINSTECH P.O. Nilore Islamabad 45650 Pakistan chuhan.pieas@gmail.com; CERIC-ERIC S.S. 14, km 163.5 in Area Science Park Basovizza 34149 Italy; ALBA Synchrotron Light Source Carrer de la Llum, 2-26 08290 Barcelona Spain

## Abstract

High temperature sintering (1200–1400 °C) has been performed on ZnO ceramics. An X-ray Absorption Fine Structure (XAFS) study shows that high sintering temperature introduces a constant amount of V_O_ and V_Zn_ defects without any significant effect on the crystal or electronic structure of Wurtzite ZnO. The combined effects of grain boundaries and voids are considered responsible for the apparent colossal dielectric constant (*ε*′) > 10^4^ at low frequency (∼10^2^ Hz) for all the sintered ZnO ceramics. The superior contact among grains of the ZnO-1200 sample enhances both the interfacial and orientational polarization of the Zn^2+^–V_O_ dipoles, which results in the increase of low and high frequency dielectric constants (*ε*′) and the corresponding dielectric loss (tan *δ*) also increases. On the other hand, high temperature sintering of ZnO at 1300 °C and 1400 °C introduces voids at the expense of reduced grain and grain boundary contact areas, thus affecting both the interfacial and orientational polarization with corresponding reduction of dielectric constant (*ε*′) and dielectric loss. Orientational polarizations due to Zn^2+^–V_O_ dipoles are suggested to remain fixed and it is the microstructure which controls the dielectric properties of high temperature sintered ZnO ceramics.

## Abstract 2

Superior grain contacts of ZnO-1200 samples enhance low and high frequency dielectric constants (*ε*′) and dielectric loss (tan *δ*).

## Graphical abstract

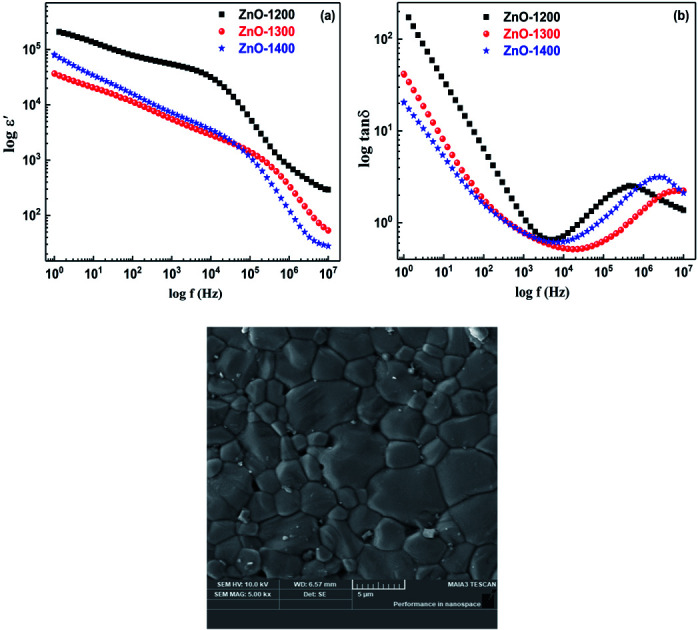

## 1. Introduction

Colossal dielectric constant (real part of permittivity *ε*′ ∼10^3^) materials are gaining much attention not only because of their fundamental academic physics but also for their potential in capacitive, high-density energy storage and electromagnetic shielding applications. Extensive studies have been carried out on high dielectric constant materials (*ε*′ > 10^4^) including ferroelectric and non-ferroelectric like CaCu_3_Ti_4_O_12_ (CCTO). Despite substantial efforts, these materials have not been fully realized towards straightforward device applications operating at frequencies of MHz and GHz. The problems of the temperature and frequency dependence of *ε*′, high magnitude of dielectric loss (tan *δ*) and difficulty in synthesizing pure phases are the main obstacles.^1–4^ Therefore, further developments towards high *ε*′ with low tan *δ* and easy to prepare materials are still essential.

Compared to the multi-component high dielectric constant materials, low cost ZnO is easier to synthesize in the pure form and is commercially available in large scale. ZnO is an academically curious direct wide band gap (*E*_g_ ∼3.37 eV) semiconductor and has attracted considerable attention due to its applications in piezotronics,^5^ super-capacitors,^6,7^ and resistive switching.^8^ Although wide range of temperature and frequency dependent dielectric behaviors of various types of ZnO nano-structures,^9^ films,^10^ and ceramics^11^ have been studied, the relative dielectric constant of pure ZnO is still quite low (<1500 at 100 Hz). The dielectric properties of ceramics are significantly influenced by the amount of porosity, its geometrical morphology and the grain boundaries.^12–14^ Controlled porosity can produce low-frequency dielectric constant ∼10^6^ in porous ceramics.^14^

In case of ZnO, role of porosity in tuning dielectric properties has been illustrated but the picture is still unclear. Li *et al.*,^15^ synthesized porous ZnO ceramics by conventional sintering under high pressure. The enhanced dielectric constant (*ε*′ ∼1.8 × 10^4^ at 100 Hz) of pure ZnO has been associated with porosity, low-density and low grain boundary resistance. On the other hand, in a recent publication Ndayishimiye *et al.*,^16^ reported fabrication of high density pure ZnO and ZnO–polydimethylsiloxane composites *via* cold sintering process. In cold sintered pristine ZnO ceramic, dielectric constants at high frequency (*ε*′ ∼140 at 10^5^ Hz) and low frequency have been associated to the dense isotropic grains and charge accumulation at grain boundaries, respectively. However, role of the porosity was totally ignored. In these reports, generally the apparent high dielectric constant of pristine ZnO was associated to Maxwell–Wagner polarization effects but the results lack proper justifications from the view point of realistic origin of the observed dielectric constant at low and high frequency sides.

The Maxwell–Wagner polarization leads to separation of charges either through inner dielectric boundary layers (*i.e.*, grain boundary regions) on a mesoscopic scale, or at the external electrode-sample interfaces on a macroscopic scale and normally considered as extrinsic effect.^17^ In the observed dielectric phenomena, the successful detachment of Maxwell–Wagner polarization from that of intrinsic grains is mandatory to consider realistic dielectric constant of the material. Therefore, present study is designed to explore the effects of microstructure (grain, grain boundaries and voids) and point defects on the dielectric properties of high temperatures sintered ZnO. Impedance Spectroscopy (IS) can separate the transport characteristics in grains, grain boundaries and at the interface.^18^ Different formalisms in IS can provide supportive information about the localized/mobile charge carriers present at different electro-active regions of sintered ZnO samples, which can be helpful in the extension of the spectrum of their applications. Therefore, a battery of characterization techniques including lab and synchrotron base X-ray diffraction (SXRD), X-ray Absorption Fine Structure (XAFS) spectroscopy, Scanning Electron Microscopy (SEM) and impedance spectroscopy have been employed to explore the possible role of sintering temperatures on the microstructure and dielectric properties of sintered ZnO ceramics. The origin of the observed dielectric constant has been explained according to the comprehensive information collected from several characterization techniques. The outcome of the present investigation might be useful to understand the nature of local structural environment and its correlation if any to the structural and dielectric properties of wide band gap semiconductors such as ZnO.

## 2. Experimental section

### 2.1 Material synthesis

High purity ZnO powder (purity 99.99% by Alfa Aesar) was used for the present study. ZnO powder was initially grounded for 45 minutes by using mortar and pastel. Subsequently, the powder was heat treated at 400 °C, 600 °C, 700 °C, 800 °C and 900 °C for 6 hours at each temperature with 30 minutes intermediate grinding. After heating at 900 °C, the powder was pressed into pellets of 10 mm diameter and thickness of 2 mm without using any binder. We further sintered ZnO pellets at 1000 °C, 1100 °C, 1200 °C, 1300 °C and 1400 °C for 10 hours. The ZnO pellets sintered at 1200 °C (ZnO-1200), 1300 °C (ZnO-1300) and 1400 °C (ZnO-1400) were finally selected for detailed analysis.

### 2.2 Material characterization

Lab base powder X-ray diffraction (XRD) measurements were performed on the sintered samples using DMAX-III diffractometer (Rigaku, Japan) operating with CuKα radiation (1.54 Å) over the angular range of 20° ≤ 2*θ* ≤ 80. High resolution Synchrotron X-ray Powder Diffraction (SXRD) data were collected at the MSPD beamline at the ALBA Synchrotron Light Source (Barcelona, Spain). The powdered samples were sealed in a 0.7 mm diameter capillary and SXRD data were collected using a continuous scanning of the MAD26 high resolution detector setup comprised of 13 Si analyzer crystals (Si 111 reflection) and scintillator/PMT detectors separated by angular offsets of ∼1.5°. Data for each pattern was collected in a 2*θ* angular range of 2–49° at a wavelength of 0.619 Å.

XAFS data at the K-edge of Zn (9659 eV) were collected at beamline 11.1 (XAFS)^19^ at the ELETTRA Synchrotron (Trieste, Italy) with the storage ring running at 2 GeV and a typical current of 300 mA. These data were collected at room temperature in transmission mode using a double crystal Si(111) monochromator. The data for Zn metal foil placed in a second experimental chamber after the sample was collected simultaneously with each sample and used for energy calibration and to check the stability of the beamline and optics system. Multiple spectra were collected for each sample (2–5 scans) and merged together to get better statics and sufficiently high signal-to-noise ratio. All the transmission spectra were collected with an integration time of 3 s per point with a variable energy step: large step (5 eV) in the first 200 eV of the spectrum, smaller step (0.2 eV) in the XANES region and a constant *k* step of 0.03 Å^−1^ in the EXAFS region.

Field Emission Scanning Electron Microscope (FESEM, TESCAN MAIA3) was used to study the surface morphology and for Energy Dispersive X-ray (EDX) analysis of the samples. Room temperature impedance spectroscopy (IS) measurements were performed on samples sintered at 1000–1400 °C in a wide frequency range (01 Hz to 10 MHz) using an Alpha-N analyzer (Novocontrol, Germany). However, temperature dependent (20 °C to 90 °C) IS measurements were performed on ZnO-1200, ZnO-1300 and ZnO-1400 ceramic pellets. To perturb the system, an AC signal of 0.2 V was used. Contacts were made on opposite sides of pellets in capacitor mode using silver paste, and cured under a tungsten lamp for 3 h. Leads were carefully checked to ensure the absence of any irrelevant resistive or capacitive coupling in measured frequency range. Fully automated WINDETA software was used for interfacing the experimental setup of the analyzer to the computer and for data acquisition. Zview software (Scribner Associates Inc., Version 2.70) was used for fitting the equivalent circuits to the impedance data. Reduced chi-squared value below 0.0006 was used as fitting criterion.

## 3. Results and discussion

### 3.1 Structural analysis

**Fig. 1:**
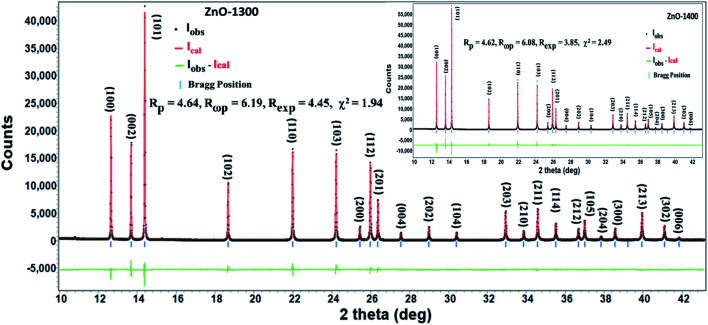
Refined XRD pattern of ZnO-1300 ceramic. Inset shows refined XRD pattern of ZnO-1400 ceramic. Black dots, red lines, horizontal bars and vertical bars represent observed data, calculated data, difference between observed & calculated data and standard Bragg positions, respectively.

Preliminary lab base XRD data of ZnO sintered at 1000–1400 °C reveals single phase hexagonal Wurtzite structure with the characteristic sharp (002) peak without any extra peak from impurity. Due to lower resolution of lab base XRD, the tiny peaks belonging to any secondary phase or impurity when they are present in small quantities are difficult to observe. However, synchrotron based XRD due to very high intensity and resolution can reveal very minor impurities. Therefore, SXRD measurements were performed on ZnO-1300 and ZnO-1400 samples and data have been analyzed by full profile Rietveld analysis method by employing Rietica software package.^20,21^ A simple fifth order polynomial background in 2*θ* along with other profile and structural parameters were refined simultaneously. Peak profile was modeled by a pseudo-Voigt peak shape function. Three parameters function described by Caglioti *et al.*^22^ was employed for the refinement of peak full width at half maximum. Instrumental peak asymmetry was modeled using a single asymmetry parameter after Howard,^23^ while preferred orientation was described by one parameter March model.^24,25^Fig. 1 and its inset show Synchrotron Powder Diffraction (SPD) patterns of ZnO-1300 and ZnO-1400 samples, respectively. The positions for all the observed diffraction peaks are in excellent agreement with the hexagonal Wurtzite ZnO structure (JCPDF#80-0075) and no additional peaks due to any impurity was observed. Crystal structure of ZnO was refined in space group *P*6_3_*mc*.^26^ The cell parameters, atomic positions and fractional site occupancy of oxygen were used as refinement parameters. The results of the refinement are presented in Table 1. Good agreement between observed and calculated diffraction peaks with reasonable goodness of fit parameter (*χ*^2^) is obtained for both ZnO-1300 and ZnO-1400 samples (Fig. 1 and its inset). The values of cell parameters obtained from the present study are in good agreement with the reported values for pure ZnO. Even high temperatures sintering (1300 °C and 1400 °C) do not show any significant effect on these parameters and all the samples maintain Wurtzite ZnO structure.^27,28^

**Table 1:** Rietveld refined parameters of ZnO sample sintered at 1300 °C and 1400 °C

Samples	Atoms	Positions			Site occupancy N*	Lattice parameters	
		*x*	*y*	*z*		*a*	*c*
ZnO-1300	Zn	0.3333	0.6667	0.0000	0.1667	3.2513	5.2077
	O	0.3333	0.6667	0.3819(4)	0.1614(7)		
ZnO-1400	Zn	0.3333	0.6667	0.0000	0.1667	3.2514	5.2086
	O	0.3333	0.6667	0.3832(4)	0.1645(4)		

In ZnO system, structural and point defects play key role in determining the crystallinity, compositional purity and dielectric properties. Although these defects do subsidize to the broadening of the XRD peaks, their accurate identification cannot be achieved *via* ordinary analysis. Therefore, we need more sophisticated tools like Extended X-ray Absorption Fine Structure (EXAFS) and X-ray Absorption Near Edge Structure (XANES) to analyze local structure around the metal centers, which may contain either undistorted or orientationally disordered ZnO_4_ tetrahedra. In the present study, the extraction of the *χ*(*k*) function was performed using the Athena program^29^ in the *k*: 2–13 Å^−1^ interval and the corresponding *k*^3^-weighted *χ*(*k*) functions were averaged.^30^ EXAFS data analysis has been performed using the Artemis software.^30^ Phase and amplitude functions were calculated by the FEFF6 code.^31^ For each spectrum, a theoretical model was designed by adding shells around the central excited atom and least-squares iterating the absorption edge energy (*E*_0_), the radial distances (*R*) and the Debye–Waller Factors (DWFs) *σ*^2^ (Å^2^). The DWFs consider both dynamic and static disorder, however dynamic disorder can be excluded as all the measurements were done at room temperature. The lattice parameters obtained from the Rietveld refinement (Table 1) were employed for generating scattering paths. Although the coordination numbers (CN) can be iterated, they were kept fixed to preset values due to their strong correlation with DWFs.^32–34^ For the Zn K-edge, the fits were performed in *R*-space in the 1–3.5 Å range. The single scattering (SS) and the most relevant multiple scattering (MS) paths involving the oxygen atoms of the first shell and the zinc atoms of the second shell, were included.

**Fig. 2:**
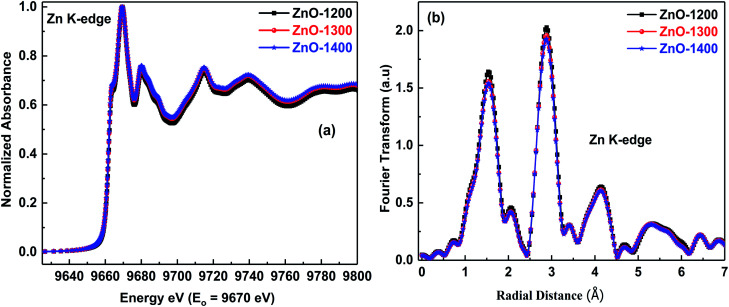
(a) Normalized XANES spectra at the Zn K-edge of the ZnO sintered at different temperatures; (b) magnitude of the Fourier transform of the same spectra.

Fig. 2(a) shows Zn K-edge normalized XANES spectra of ZnO-1200, ZnO-1300 and ZnO-1400 samples. These normalized spectra were obtained by subtracting the smooth pre-edge background from the experimental spectra, and taking the edge jump height as unity; further details of the normalization procedure can be found elsewhere.^35,36^ A preliminary investigation of the Fig. 2(a) reveals that there is no change in the Zn oxidation states even after sintering at different temperatures and the features of absorption edges are the same. It is also observed that there is no appearance of any pre-edge feature in all the spectra, suggesting the probabilities of electronic transitions from core levels to bound states of Zn atoms are absent.

In order to understand if sintering temperatures can have any influence on the Zn local environment, detailed investigation of the Zn K-edge EXAFS signal was performed. Fig. 2(b) shows comparison of the magnitude of the Fourier transforms of the Zn K-edge spectra of the ZnO sintered at different temperatures. The peak at ∼1.5 Å is due to the first shell of oxygen atoms related to the position of the Zn–O bonding distance, and the second peak contains contribution of Zn–Zn, Zn–O (second shell) and MS. Generally, magnitude of the Fourier transforms of the XAFS signal is related to the coordination number. It is evident from Fig. 2(b) that intensity of the first shell peak almost remains unchanged when samples are sintered at 1300 °C and 1400 °C temperatures thereby suggesting no appreciable change in the coordination numbers.

Fourier transforms (both experimental and fitted model) of the Zn K-edge spectra of the sintered ZnO samples are shown in Fig. S1 in ESI† and best-fitted results are presented in Table 2. We can see from Table 2 that the first shell interatomic distances are comparable to those reported for pure ZnO ceramic.^37,38^ It seems that high temperature sintering does not influence the oxygen radial distances in a significant way. On the other hand, for all the sintered samples of the ZnO, the second shell radial distance is slightly larger than the reported values of undoped ZnO ceramic.^37,38^ The observed DWFs of second shells are also higher compared to other shells but remain almost same for temperatures of 1300 °C and 1400 °C. High DWFs generally indicates a high degree of local disorder around the absorbing atoms and suggest introduction of atomic vacancies.^39^ Present study suggests that high temperature sintering of ZnO at 1200 °C introduces local disorder that remains unchanged on further sintering temperatures of 1300 °C and 1400 °C. In ZnO, oxygen vacancy (V_O_) and zinc vacancy (V_Zn_) are respectively considered to introduce shrinkage and expansion in the lattice parameters^40^ and presence of V_Zn_ is also reported in the second coordination shell.^41^ Therefore, observed increase in the DWFs and radial distances of the second shell Zn–Zn atoms suggests introduction of local disorder around Zn absorbing atoms due to possible creation of V_Zn_. Further, the second shell DWFs remain unchanged for all the samples of ZnO sintered from 1200 °C to 1400 °C signifying that the amount of V_Zn_ concentration remains unchanged and high sintering temperature has very little effect on the overall electronic structure.

**Table 2:** Crystallographic data and structural parameters as obtained from the *R*-space fit of the ZnO sample sintered at different temperatures

Samples	Shells	*N*	S^2^_O_	*σ* ^2^ (Å^2^)	*R*_eff_ (Å)	*R* (Å)
ZnO-1200	Zn–O	4	0.713	0.0036 ± 6	1.9788	1.9685 ± 9
	Zn–Zn	12	0.713	0.0091 ± 7	3.2102	3.2442 ± 7
	Zn–O–Zn	24	0.713	0.0077 ± 7	3.5941	3.3209 ± 1
	Zn–O	9	0.713	0.0055 ± 4	3.8101	3.7694 ± 9
ZnO-1300	Zn–O	4	0.712	0.0041 ± 6	1.9788	1.9660 ± 1
	Zn–Zn	12	0.712	0.0094 ± 6	3.2102	3.2385 ± 2
	Zn–O–Zn	24	0.712	0.0087 ± 8	3.5941	3.3549 ± 1
	Zn–O	9	0.712	0.0063 ± 5	3.8101	3.7651 ± 5
ZnO-1400	Zn–O	4	0.704	0.0040 ± 6	1.9788	1.9657 ± 9
	Zn–Zn	12	0.704	0.0094 ± 6	3.2102	3.2384 ± 5
	Zn–O–Zn	24	0.704	0.0091 ± 7	3.5941	3.3534 ± 7
	Zn–O	9	0.704	0.0060 ± 4	3.8101	3.7656 ± 9

### 3.2 Microstructural analysis

**Fig. 3:**
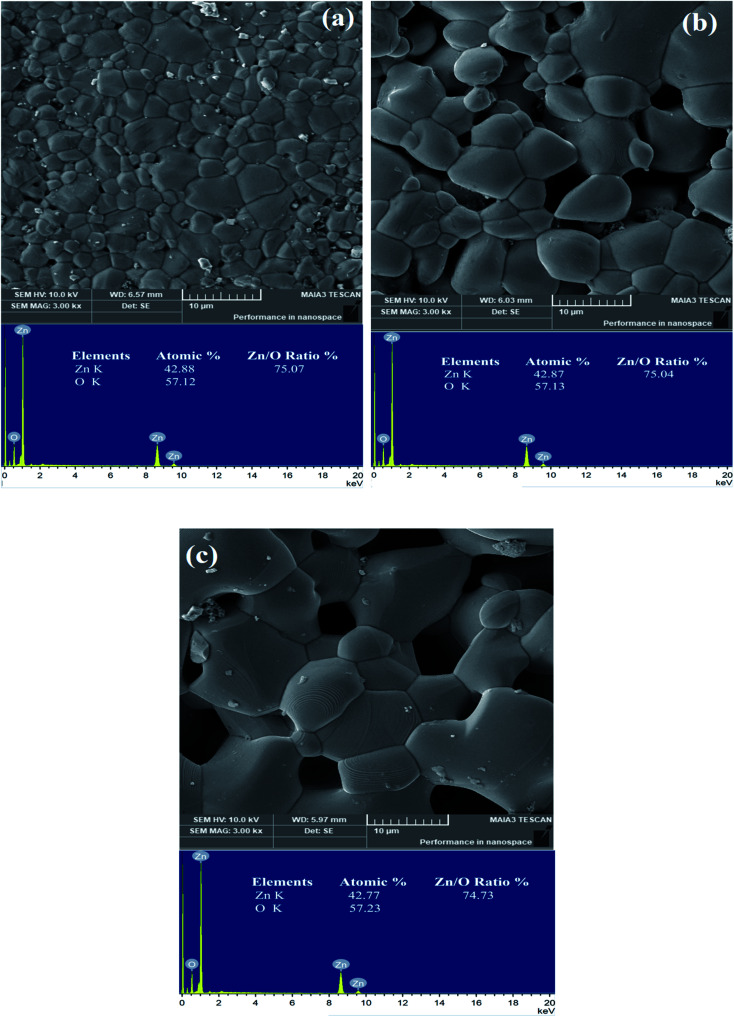
(a) SEM micrographs of (a) ZnO-1200 sample, (b) ZnO-1300 sample and (c) ZnO-1400 sample. EDX images along with Zn/O atomic ratio (%) are also presented in lower inset of each figure.

In order to investigate morphology and grain growth of the ZnO samples sintered at different temperatures, detailed SEM analysis has been performed as shown in Fig. 3(a–c). The ZnO-1200 sample shows very compact structure with broad inhomogeneous distribution of well refined and fully developed crystallite sizes in the range of ∼1–10 μm (Fig. 3(a)). The micrograph also demonstrates very good grain-to-grain contact with minimum amount of isolated voids. The intimate contact between ZnO grains apparent in the SEM image should produce promising electron transport properties in this sample. Subsequent ZnO sintering at higher temperature of 1300 °C significantly introduces voids and grain growth compared to the ZnO-1200 sample having crystallite sizes in the range of 3–15 μm (Fig. 3(b)).

During 1300 °C heating process, relatively bigger ZnO crystallites easily form large structure due to diffusion of small crystallite around them, while the voids between ZnO crystallites are difficult to remove thereby forming open void structure around the grain boundaries. The increased amount of open voids seems to be associated to the decreasing grain-to-grain connections due to steady decrease in grain boundary contact area. Further ZnO sintering at 1400 °C temperature considerably changes microstructure and produce crystallite size in the range of 3–20 μm as shown in Fig. 3(c). It seems that due to increase in grain size of ZnO-1400 sample, it shows relatively lower amount of voids than that of ZnO-1300 sample. To find out the possible existence of metal cluster and foreign elements at grain and grain boundaries, we have performed EDX analysis at different areas of the grain and grain boundaries of all the sintered ZnO samples (lower insets of Fig. 3(a–c)). The EDX spectra reveal only zinc and oxygen atoms belonging to the native ZnO matrix and Zn/O atomic ratio ∼75% remains fixed for all the sintered samples. The dust like particles on the surface of the sintered ZnO ceramics shown in each micrograph also belongs to the ZnO and excludes the possibility of impurity atoms. Within the detection limit of EDX, we do not observe any foreign atom or metal clustering in the sample.

### 3.3 Impedance analysis

In most of the oxide ceramics, grain boundaries are more resistive than grains due to non-stoichiometric distribution of oxygen and dangling bonds, which act as the carrier's traps and produce a barrier layer.^42^ Furthermore, electrode contact impedances have much higher capacitances ∼10^−7^ to 10^−5^ F compared with ∼10^−10^ to 10^−8^ F and ∼10^−12^ F capacitances of grain boundaries and bulk, respectively.^43,44^ AC electrical response in polycrystalline materials usually relates the effects of electrode contact, inter-grain (grain boundaries) and intra-grain (bulk) effects at low, intermediate and high frequencies, respectively.^18,43^ This makes the visualization and representation of the properties of a sample an easy process without the need to exclude sample-electrode contact impedances; however, complete analysis of the sample-electrode impedances is mandatory to fully filter intrinsic and extrinsic behaviors of the material.

**Fig. 4:**
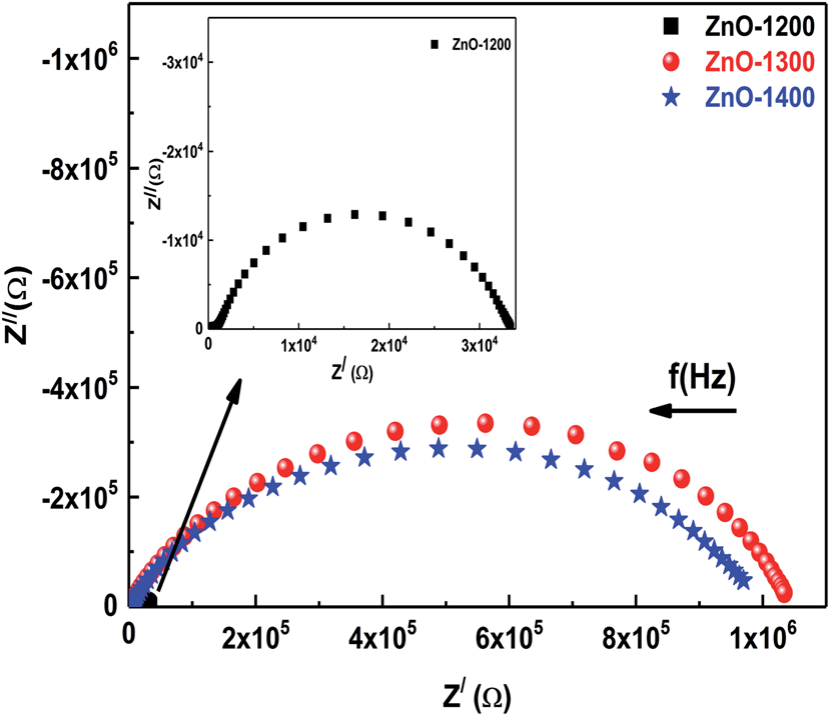
Impedance plane plots (*Z*′′ *vs. Z*′) of ZnO sintered at 1200–1400 °C. Inset shows impedance plane plot for ZnO-1200. Right arrow shows increase in frequency from right to left.

The prime objective of the impedance analysis is to explore the most suitable equivalent circuit to characterize the data sets. Fig. 4 shows impedance plane plots *Z*′ *vs. Z*′′ as a parametric function of frequency for ZnO ceramics sintered at different temperatures. The solid symbols represent the experimental data and the frequency increases from right to left as shown by the right hand side arrow. The diameter of semicircular arc on *Z*′-axis is the resistance of involved micro-structural component, whereas total resistance of a system under investigation is the sum of diameters of all the arcs appear in impedance plane plots.^18,43^ The ZnO-1200 sample shows the smallest diameter of the semicircular arc, hence lowest resistance. A remarkable increase in the diameters of the semicircular arcs is observed when sintering temperature of the ZnO is increased to 1300 °C and 1400 °C. Increase in the overall resistance upon sintering suggests breaking of the intimate contact between ZnO grains due to introduction of the voids, which is in agreement with the SEM analysis discussed previously. Subsequent sintering at 1400 °C displays a slight decrease in resistance possibly due to grain growth.

Collection of typical impedance data for three samples reveals two similar characteristics. The first common feature in all three samples is the depressed semicircular arc at high frequency side whose width is larger than its height 
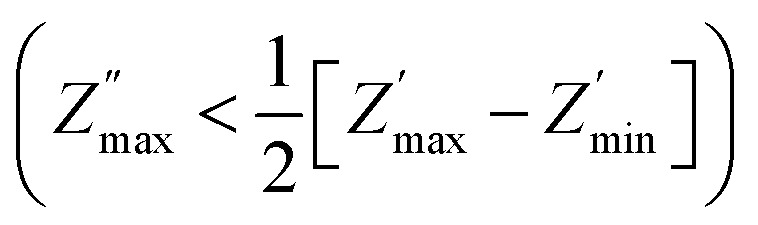
, where 
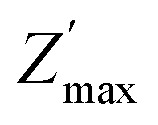
 and 
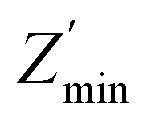
 are the intercepts of the arc with the real axis at low and high frequencies, respectively.^18,43^ Due to the presence of defects, structural stresses and distributed elements in the system, the center of the semi-circle is displaced below the real axis. This compression in the semicircular arcs is addressed by depression angel and is related to the deviation of the center of the semicircular arc below the real axis. By employing Zview software, depression angles of the small semicircular arc at higher frequency side worked out to be ∼49°, 28° and 38° for ZnO-1200, ZnO-1300 and ZnO-1400 samples, respectively. Second common feature in the impedance plane plots is the larger semicircular arc at low frequency side. The values of the depression angles of large low frequency semicircular arcs are 13°, 25° and 29° for the ZnO-1200, ZnO-1300 and ZnO-1400 samples, respectively.

Relatively higher values of the depression angles show the presence of more distributed elements with diverse relaxation times due to the existence of micro-structural heterogeneities and defects.^18^ To address the heterogeneity of the samples, the constant phase element ‘CPE’ is generally used instead of capacitance to account the non-ideal behavior (*i.e.*, depression of semicircular arc center below the *Z*′-axis) to fit the impedance plane plot data. The CPE can be deconvoluted into resistive and capacitive components, whose relative contribution is given by the parameter “*n*”, which is a measure of deviation from the ideal behavior having a value of 0 for pure resistive and 1 for pure capacitive behavior. The capacitance of a CPE is given by the relation *C* = *R*^(1−*n*)/*n*^(CPE)^1/*n*^; where *C* and *R* are capacitance and resistance of the associated component.^18^ A specific feature of a CPE is that it provides a power law dependence on frequency scale to both the resistive and capacitive components. The CPE is frequently added subsequently with (i) a resistance *R*, which relates to the limiting low frequency or DC resistance of the sample and (ii) a capacitance *C* that links to the limiting high frequency permittivity.^45,46^

**Fig. 5:**
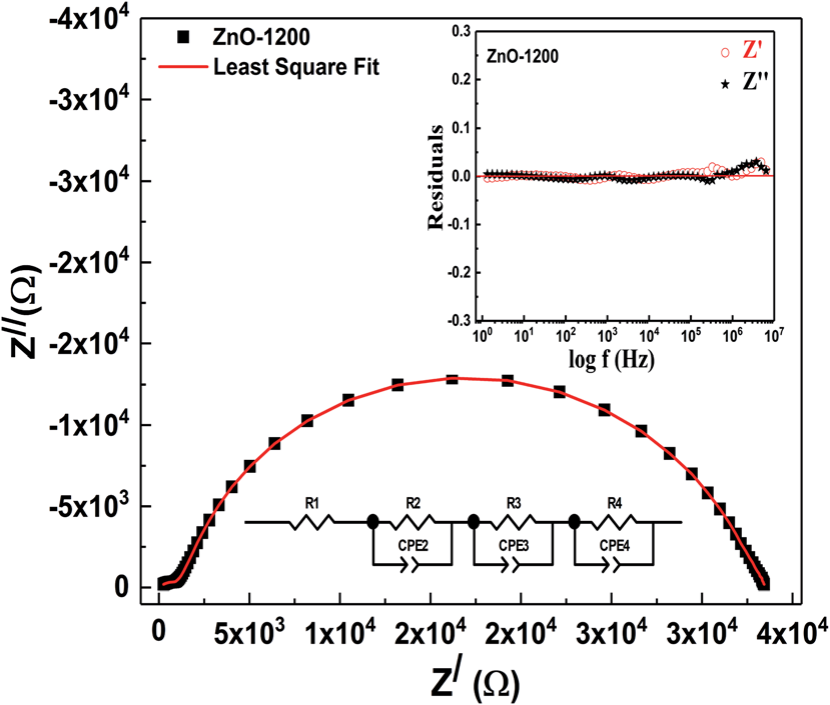
Impedance plane plots (*Z*′′ *vs. Z*′) of ZnO-1200 sample. The symbols and solid lines represent the data points and fitted lines, respectively. The equivalent fitted model circuit is presented in lower inset. Upper right inset shows residuals between experimental and fitted data for the same sample.

Physical characteristics of the sample have been evaluated by circuit analysis to make assignments of the several circuit elements to electrical features of the sample. In ZnO and other ceramic oxides, the grain boundary resistance is generally higher in comparison to the grains due to segregation of point defects (*i.e.*, oxygen vacancies, zinc interstitial and zinc vacancies) at grain boundaries.^47–49^ In addition, voids exist in the form of three-dimensional pockets around the grain boundaries. The high surface area of the voids provides chance for the defects to be distributed and creates obstruction to the flow of charge carriers by reducing the conduction path and compressing current lines, analogous to the effect of impurities at grain boundaries.^48,50^ A relationship of the two commonly observed features (small and large semicircular arcs) has been made based on the magnitudes of the associated capacitances. Thus; the high frequency arc is endorsed to the bulk response and the low frequency arc to grain boundary impedance. In the view of discussion above, an equivalent circuit model along with least square fitting data provides a reasonable fit to the impedance data set of ZnO-1200 sample as shown in Fig. 5. The component *R*_1_(*R*_2_CPE_2_) connected in series best fit to the high frequency arc and deviation from the ideality of the distorted semicircle is the signature of the element CPE. The non-ideality is apparent by the relatively higher value of the depression angel (49°) and it has also been observed that high frequency arc does not pass though the origin for ZnO-1200 sample.

In the impedance plane plots, sometimes the semicircular arc does not pass through the origin due to other arcs having higher relaxation frequencies beyond the measurement limit,^18,51^ which can be better resolved by other formalisms. Therefore, the resistance *R*_1_ in the equivalent circuit is assigned to account for the shift in the origin at high frequency along the *Z*′-axis (*R*_S_) for ZnO-1200, ZnO-1300 and ZnO-1400 samples. The resistance *R*_2_ is associated to the grains (*R*_G_). Relatively distorted semicircle at lower frequency side shows at least two different relaxation frequencies during fitting procedure. Moreover, from the SEM analysis, we also observe inhomogeneous distribution of crystallite sizes and voids in all the ZnO ceramic samples. Therefore, resistances *R*_3_ and *R*_4_ of the semicircular arcs at intermediate and lower frequencies are assigned to the resistances of conventional (intrinsic) grain boundaries (*R*_GB1_) and un-conventional (extrinsic) grain boundaries (*R*_GB2_), respectively. Unconventional grain boundaries (*R*_GB2_) are referred here as a combination of grain boundaries and voids that are present very close to each other. Final equivalent circuit for ZnO-1200 sample turns out to be (*R*_S_)(*R*_G_CPE_G_)(*R*_GB1_CPE_GB1_)(*R*_GB2_CPE_GB2_). The parameters *R*_S_, *R*_G_, *R*_GB1_, *R*_GB2_, CPE_G_, CPE_GB1_, and CPE_GB2_ were obtained for each ZnO sample sintered at different temperatures by fitting the impedance plane plots (∼5% fitting error) and are presented in Table 3.

**Table 3:** Zview fitted parameters for ZnO-1200, ZnO-1300 and ZnO-1400 samples

Fitting parameters	ZnO-1200	ZnO-1300	ZnO-1400
*R*_S_	1.27 × 10^2^	9.81 × 10^2^	1.83 × 10^3^
*R*_G_ (Ω)	5.52 × 10^2^	5.97 × 10^2^	1.74 × 10^3^
*R*_GB1_ (Ω)	2.83 × 10^4^	1.66 × 10^5^	9.50 × 10^5^
*R*_GB2_ (Ω)	4.95 × 10^3^	8.78 × 10^5^	4.08 × 10^4^
*R*_T_ (Ω)	3.39 × 10^4^	1.05 × 10^6^	1.39 × 10^5^
*C*_G_ (F)	1.10 × 10^−10^	5.27 × 10^−11^	1.53 × 10^−11^
*C*_GB1_ (F)	6.94 × 10^−9^	1.40 × 10^−9^	2.23 × 10^−9^
*C*_GB1_ (F)	7.61 × 10^−8^	1.72 × 10^−9^	4.47 × 10^−9^
*n*_G_	0.84	—	—
*n*_GB1_	0.90	0.74	0.69
*n*_GB2_	0.39	0.77	0.97
*f*_Rex-G_ (Hz)	3 × 10^6^	5 × 10^6^	6 × 10^6^
*f*_Rex-GB1_ (Hz)	1 × 10^3^	7 × 10^2^	8 × 10^1^
*f*_Rex-GB2_ (Hz)	4 × 10^2^	1 × 10^2^	9 × 10^2^

Similarly, an equivalent circuit (*R*_1_)(*R*_2_C_2_)(*R*_3_CPE_3_)(*R*_4_CPE_4_) along with least square fitting data shown in the corresponding Fig. S2 and S3 in ESI† provides a reasonable fit to ZnO-1300 and ZnO-1400 ceramics and best fit parameters are listed in Table 3. With usual meanings of *R*_1_, *R*_2_, *R*_3_ and *R*_4_ discussed above, final circuit becomes (*R*_S_)(*R*_G_*C*_G_)(*R*_GB1_CPE_GB1_)(*R*_GB2_CPE_GB2_). In all the cases, excellent agreement is seen between experimental data and fitting model over the entire frequency range. The alternative methodology to check quality of the fit is to plot the residuals between experimental and fitted data as a function of frequency. The upper right insets of Fig. 5, S2 and S3 in ESI† show that residuals between experimental and fitted data fluctuate around zero, except at the high frequency limit, where the experimental data is prone to larger errors, but still remains close to zero. In the impedance analyzers, the high-frequency variation in residuals is normally caused due to inductance of the coaxial cable, known to occur at high-frequency/low-resistance values.^52^ Moreover, for all the ZnO samples, our attempt to fit the data with three components by dropping *R*_1_ (*i.e. R*_S_) from the master circuit resulted in significantly inferior chi-squares values than those obtained by the four components fitting including *R*_1_.

The result presented in Table 3 shows that with increase in sintering temperature of ZnO ceramics, all the three components *R*_S_, *R*_G_, *R*_GB1_ progressively increases. It has been observed that when the amount of voids is negligible and all the grains are connected in three dimensions, the grain and grain boundary resistances are low as is the case of ZnO-1200 sample. However, with the increase in sintering temperature to 1300 °C, the amounts of voids increases and some of the grains become isolated. This results in the increase of the effective surface resistance of both the grain interior (*R*_G_) and grain boundary resistance (*R*_GB1_) adjacent to the grains.^53^ Similarly unconventional grain boundary resistance (*R*_GB2_) also increases due to introduction of voids at the expense of reduced grain boundary contact area around the voids. Three-dimensional pockets of the voids around the grain boundaries offer high surface area to the defects to be distributed and damage the conduction path of the charge carriers thereby increasing resistance to the transport of charge carriers.^48,50^ For ZnO-1400 sample, we observe reduction in the values of *R*_GB2_, which is suggested due to conversion of open voids to close voids due to grain growth as shown by Fig. 3(c).

Up till now, we have discussed impedance plane plots and established the link among possible electroactive regions and best fitted capacitances and resistances. Impedance complex plane plot Z* (*i.e.*, *Z*′′ *vs. Z*′) is a good method to distinguish bulk and grain boundary resistances. However, Z* plot on linear scale offers unnecessary weighting to the largest resistances in a sample and effectively ignores from view the conductive grain cores surrounded by resistive grain boundaries.^54^ A comprehensive analysis of impedance data that sidesteps such weighting is presented by demonstrating the same impedance data in at least two of the four formalisms: impedance Z*, admittance Y*, electric modulus M* and permittivity ε*.

**Fig. 6:**
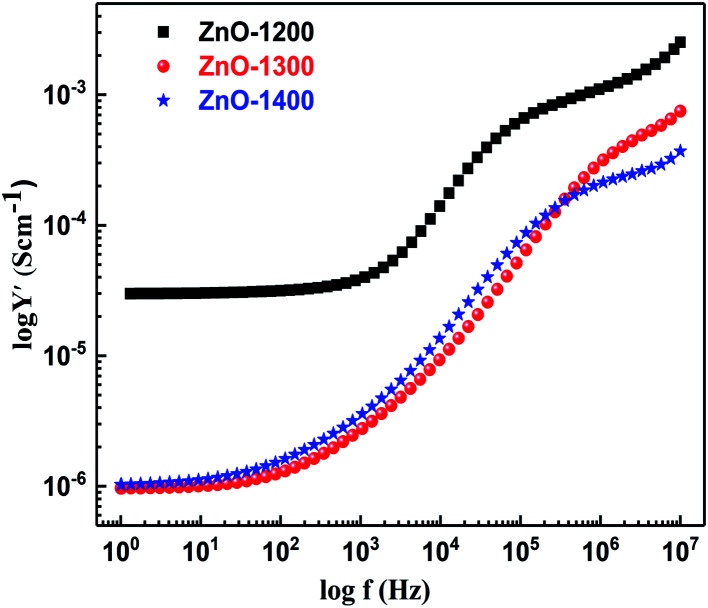
Electrical admittance as a function of frequency for ZnO sample sintered at 1200–1400 °C.

Log *Y*′ *vs.* log *f* plot provides an equivalent credit to the different conducting elements and in particular emphasizes the existence of a high frequency dispersion modeled by the bulk constant phase element (CPE).^54^Fig. 6 shows admittance or conductivity plot (log *Y*′ *vs.* log *f*) for ZnO sintered samples. Typical admittance spectra for all the samples show two different regions *i.e.* plateau region and the frequency dependent region ∼10^3^ Hz. The plateau region is frequency independent and is referred as DC conductivity, which arises from the hopping of carriers between strictly localized states. The two frequency dependent regions at higher frequencies include the conductivity of the easier hops. For all the sintered samples, there is an evidence of second and third series CPE components in the equivalent circuit models corresponding to the two frequency dependent regions of log *Y*′ *vs.* log *f* spectra. The presence of a CPE in the equivalent circuit represents the power law dispersion. Comparatively, ZnO-1200 sample indicates higher magnitude of conductivity with a well-defined DC limit signifying the formation of an electronically conducting path in agreement with SEM results where we found excellent intimate contact among grains. For ZnO-1300 and ZnO-1400 samples, the region of low frequency DC limit shrinks due to expansion in frequency dependent region, which might be related to the combined effects of dissipative process of dipole reorientation and geometrical heterogeneity around the dipoles (*i.e.*, grain and grain boundaries).

**Fig. 7:**
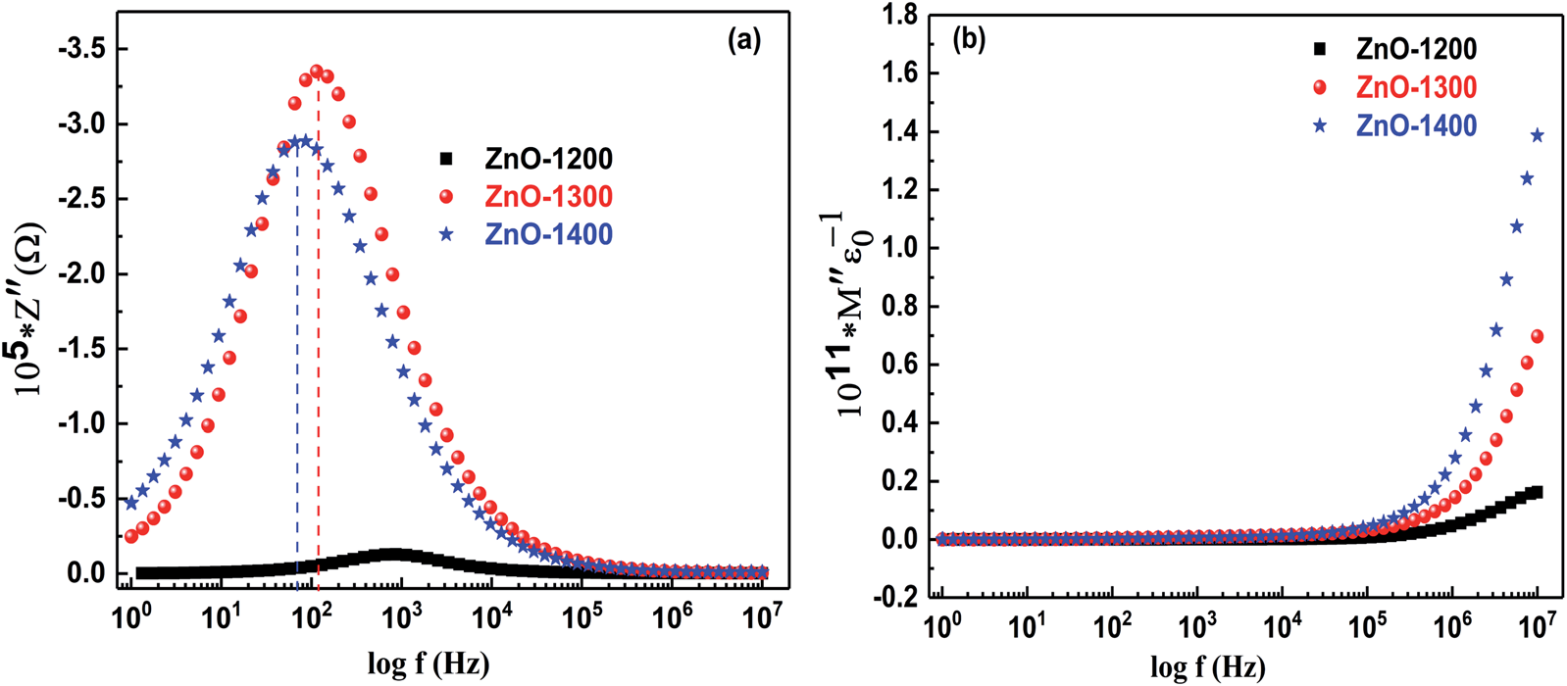
Bod plots for ZnO sample sintered at 1200–1400 °C in (a) impedance (*Z*′′ *vs.* log(*f*)) and (b) modulus (*M*′′ *vs.* log(*f*)) formalisms.

Generally, together with the impedance, modulus formalism is used to separate grain and grain-boundary effects to distinguish the microscopic processes responsible for localized and long-range conductions. The simultaneous presentation of *Z*′′ *vs.* log *f* and *M*′′ *vs.* log *f* is helpful to see whether the main resistance shown by the largest arc in *Z*′′ *vs. Z*′ plots or the largest peak in *Z*′′ *vs.* log *f* plots specifies the sample bulk. Occurrence of relaxation peak in the loss spectrum (*Z*′′ *vs.* log *f*) is the indication of the current dissipation and is related to the most resistive part of the material. Similarly, modulus formalism (*M*′′ *vs.* log *f*) is associated with the bulk properties of the material and demonstrates a maximum at a characteristic frequency in the conduction process of the relevant phase.^18^ Therefore, to have an insight into the relaxation process and conduction mechanism, we have studied both impedance (*Z*′′ *vs.* log(*f*)) and modulus (*M*′′ *vs.* log(*f*)) formalisms as presented in Fig. 7(a and b).

All the sintered samples of ZnO show only one broad relaxation peak in the *Z*′′ *vs.* log(*f*) spectra as presented in Fig. 7(a). The relaxation peak ∼10^3^ Hz for ZnO-1200 sample gradually shifts towards lower frequency at and down to 10^2^ Hz for ZnO-1300 and ZnO-1400 samples, respectively. This shift in relaxation peak towards lower frequency side is related to the introduction of micro-structural heterogeneities (*i.e.* grain boundaries and voids) upon sintering. The associated dipoles present at the micro-structural heterogeneities face difficulty in flipping their poles and therefore, show their relaxation at much lower frequency side. The relaxation frequency (*f*_Rex_) of the charge carries present at micro-structural heterogeneities were estimated by *f*_Rex_ = 1/2π*RC*, where *R* and *C* are the resistance and capacitance of individual components, respectively and results are presented in Table 3. From Table 3, it is clear that difference in relaxation frequencies of the dipoles present at conventional and unconventional grain boundaries (*i.e.*, *f*_Rex-GB1_ and *f*_Rex-GB2_) is only of an order of magnitude for all the sintered samples of ZnO. This close proximity of values of relaxation frequencies of *f*_Rex-GB1_ and *f*_Rex-GB2_ makes it hard to spread the competing phases and results in the occurrence of a broad loss peak at low frequency side in the *Z*′′ *vs.* log(*f*) formalism.^42,51^ On the other hand, absence of loss peak in the high frequency region may be related to the dominancy of dipoles in the conduction process whose relaxation may occur out of available experimental frequency range. The *M*′′ *vs.* log(*f*) spectra presented in Fig. 7(b) for all the ZnO sintered samples do not show any peak and it seems that relaxation peak may be present beyond measured frequency range. In the present study, the high frequency peak associated with relaxations of the dipoles present at the intrinsic grains is not resolved by both impedance (*Z*′′ *vs.* log(*f*)) and modulus (*Z*′′ *vs.* log(*f*)) formalisms. However, for all three samples, high frequency dipolar relaxation peak is well resolved in the dielectric loss (tan *δ*) spectrum and will be discussed in the upcoming section.

**Fig. 8:**
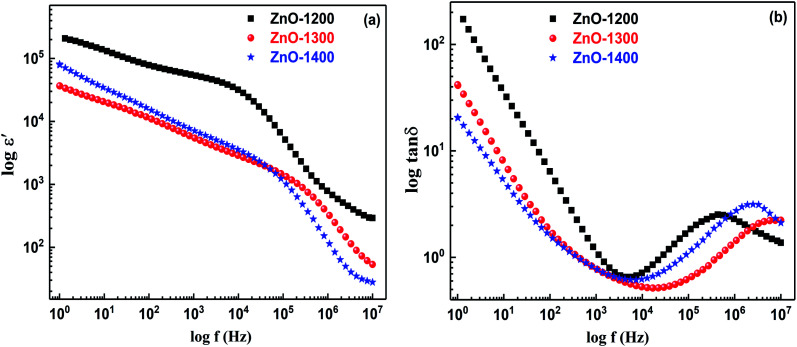
Bod plots for ZnO sample sintered at 1200–1400 °C in (a) log *ε*′ *vs.* log(*f*) and (b) log tan *δ vs.* log(*f*) formalisms.

Fig. 8(a) shows room temperature dielectric constant (*ε*′) against frequency for all three samples sintered at different temperatures. The dielectric response for all the samples clearly reveals at least one dispersion region. Different surrounding environments for carriers present at grain boundaries and close to the voids may produce more than one relaxation process. Those electroactive regions having alike or comparable relaxation frequencies for charge carriers involved in conduction and are difficult to resolve; therefore, dispersion in dielectric constant at low frequency (<10^4^ Hz) is not resolved in spectrum due to very close relaxation times of the charge carries present at their related heterogeneity.

Fundamentally, four kinds of polarization are described, namely interfacial, dipolar, atomic and electronic.^55^ Interfacial polarization has a significant role in tuning the dielectric characteristics of materials below 10^2^ Hz where grain boundaries are more dominant.^42,56^ In the present study, at lower frequency (∼10^2^ Hz), the magnitude of *ε*′ > 10^4^ for all the sintered samples fulfills the definition of colossal dielectric constant materials.^3^ ZnO-1200 sample shows larger value of *ε*′ at both low and higher frequencies than that of ZnO-1300 and ZnO-1400 samples. The observed dielectric response below 10^2^ Hz and above 10^5^ Hz can be attributed to the extrinsic (interfacial polarization; grain boundaries and voids) and intrinsic (grain interior) effects, respectively. In the pristine ZnO, polarization is generally governed by orientational polarization due to association of the Zn^2+^ with oxygen vacancies, which have limited dipole moment.^57,58^ In the present study, all the samples were heat treated at very high temperatures (1200–1400 °C) and XAFS study shows that amount of V_O_ and V_Zn_ concentrations more or less remains the same in all the samples. Therefore, orientational polarization due to the Zn^2+^–V_O_ dipoles remains fix and it is the microstructure, which controls the overall dielectric constant in the whole frequency range of all the sintered ZnO ceramics.

High magnification SEM micrograph of the ZnO-1200 sample depicts very dense microstructure and good grain to grain contacts with minimum number of voids as shown in Fig. S4 in ESI.† The effective intimate contact between two consecutive grains produces less grain boundary resistance as evidenced by impedance analysis. Therefore, high frequency (>10^5^ Hz) dielectric constant may arises mainly due to the orientational polarization of the Zn^2+^–V_O_ dipoles present at the grain interior and can be considered as an intrinsic effect. As sintering temperature increases to 1300 °C and 1400 °C, dispersion in dielectric constant extends and shifts towards higher frequencies thereby suggesting good grain growth, which is in agreement with SEM results. Increased grain growth provides more defect free grain interiors by removing trapped charges and therefore Zn^2+^–V_O_ related dipoles relax more freely at high frequency side.

Alteration in the tangent loss (tan *δ*) with respect to frequency for all the ZnO sintered samples are presented in Fig. 8(b). It is observed that ZnO-1200 sample show greater tan *δ* values compared to other samples in the measured frequency range. Furthermore, each spectrum possesses at least one dielectric loss peak showing at least one relaxation process in the system. The tan *δ* peak in high frequency region >10^5^ Hz is ascribed to the relaxation process associated with Zn^2+^–V_O_ dipole present in grains. It has also been observed that as sintering temperature increases from 1200 °C to 1400 °C, the tan *δ* peak shifts towards higher frequency side due to introduction of grain growth. Grain growth promotes a defect-free structure inside the grains and associated dipoles involved in the polarization show their tan *δ* peak at high frequency region. Therefore, in sintered ZnO samples, tan *δ* peak above 10^5^ Hz is credited to relaxation of dipoles present in grain interior and can be considered as an intrinsic loss effect. The extracted dielectric constant (*ε*′) and dielectric loss (tan *δ*) values at different frequencies for all the sintered samples of ZnO are presented in Table 4.

**Table 4:** Dielectric parameters for ZnO-1200, ZnO-1300 and ZnO-1400 samples

Samples	Dielectric constant (*ε*′)				Dielectric loss tan *δ*			
	10^2^ (Hz)	10^5^ (Hz)	1 MHz	5 MHz	10^2^ (Hz)	10^5^ (Hz)	1 MHz	5 MHz
ZnO-1200	1.9 × 10^4^	1.2 × 10^3^	1.8 × 10^2^	90.6	9.2	1.9	2.2	1.6
ZnO-1300	2.5 × 10^3^	3.2 × 10^2^	78.0	21.7	2.0	0.7	1.5	2.2
ZnO-1400	3.5 × 10^3^	2.5 × 10^2^	27.5	08.5	1.6	1.2	2.7	2.8

At low frequency (10^2^ Hz), the observed dielectric constant *ε*′ ∼1.9 × 10^4^ for ZnO-1200 sample presented in Table 4 is comparable to the highest reported values of *ε*′ ∼1.8 × 10^4^ at 10^2^ Hz by Li *et al.*,^15^ for the pristine porous ZnO ceramic sintered at 1050 °C under high pressure. However, at high frequency side (above 10^5^ Hz), the interesting feature of ZnO-1200 sample is its comparable dielectric constant (*ε*′) with low dielectric loss (tan *δ*) than the reported ones^15,16^ along with good temperature stability as shown in Fig. S5(a and b) in ESI.† Contrary to previous observations,^15,16^ present study shows that at high frequency, improved, stable and realistic dielectric constant with low dielectric loss values of ZnO-1200 ceramic excludes the role of voids; instead, defect free grain interiors with excellent intimate contacts among them promote enhanced orientational polarization of the Zn^2+^–V_O_ dipoles. The superior contact among grains boost both the interfacial and orientational polarization, which result in the increase in low and high frequency dielectric constant (*ε*′) and corresponding dielectric loss also increases. On the other hand, high temperature sintering of ZnO at 1300 °C and 1400 introduces voids at the expense of reduced grain and grain boundary contact areas, thus damage to the both interfacial and orientational polarization resulting in the corresponding reduction of dielectric constant (*ε*′) and dielectric loss.^59–61^

Although at lower frequency (∼10^2^ Hz) all the sintered ZnO ceramics show colossal *ε*′ > 10^4^, the observed dielectric response is unrealistic and related to combined effects of grain boundaries and voids. This phenomenon is also evident by the unstable temperature dependent dielectric constant (*ε*′) and dielectric loss (tan *δ*) at lower frequency side, which becomes stable at higher frequencies of ≥10^5^ Hz for the same ZnO-1200 ceramic as presented in Fig. S5(a and b) in ESI.† Room temperature impedance data for ZnO ceramic sintered at 1000 °C and 1100 °C has also been collected in order to evaluate low sintering temperature effects on dielectric constant *ε*′ and tan *δ* values. The *ε*′ and tan *δ* data for ZnO sintered at 1000–1400 °C (Fig. S6(a and b) in ESI†) shows that although 1000 °C and 1100 °C sintered samples have lower tan *δ* values at higher frequencies of 10^5^ Hz, corresponding *ε*′ is quite low. Compared to 1200 °C sintering temperature, it is expected that at lower sintering temperatures of 1000 °C and 1100 °C, ZnO grains are not fully developed and are more coarsed, which cannot enhance both the interfacial and orientational polarizations and result in the lower dielectric constant (*ε*′) and dielectric loss in the entire measured frequency region. This implies that among different sintered samples, pristine ZnO ceramic sintered at 1200 °C still produces high *ε*′ with low tan *δ* values.

## 4. Conclusions

We have performed systematic SXRD, XAFS, SEM and detailed impedance spectroscopy on high temperatures sintered (1200–1400 °C) ZnO ceramics to understand the possible role of sintering temperatures on the crystal structure, microstructure and dielectric properties of sintered ZnO ceramics. High sintering temperature creates constant amount of V_O_ and V_Zn_ defects without modifying structure of Wurtzite ZnO as revealed by XRD and XAFS analyses. In all the sintered ZnO ceramics, the observed dielectric response below 10^2^ Hz and above 10^5^ Hz can be attributed to the extrinsic (interfacial polarization; grain boundaries and voids) and intrinsic (grain interior) effects, respectively. From the microscopic view point, interfacial and orientational polarization of the Zn^2+^–V_O_ dipole tune both dielectric constant (*ε*′) and dielectric loss ability (tan *δ*) at low and high frequency sides respectively. These microscopic entities are highly correlated with microstructure of the sintered samples. Unrealistic colossal dielectric constant *ε*′ > 10^4^ at low frequency (∼10^2^ Hz) has been associated to the combined effects of grain boundaries and voids. While at high frequency (>10^5^ Hz), stable and realistic dielectric constant with low dielectric loss value of pristine ZnO-1200 sample is originated by excellent intimate contacts of the defect free grain interiors. The combined microscopic and spectroscopic approach towards realistic dielectric response will open up new avenues from the fundamental understanding and practical viewpoints.

## Conflicts of interest

There are no conflicts to declare.

## Supplementary Material

RA-010-D0RA04273K-s001
