# Supplementary material for: Realistic dielectric response of high temperature sintered ZnO ceramic: a microscopic and spectroscopic approach
Source: RSC Adv. 2020 Aug 18;10(51):30451–62. doi: 10.1039/d0ra04273k (PMC9056371; doi:10.1039/d0ra04273k)
Supplement: RA-010-D0RA04273K-s001 [file RA-010-D0RA04273K-s001.pdf]

Electronic Supplementary Material (ESI) for RSC Advances.
This journal is © The Royal Society of Chemistry 2020

Electronic supplementary information (ESI)

Realistic Dielectric Response of High Temperature Sintered ZnO Ceramic: A Microscopic and Spectroscopic Approach

Sidra Ibadat[1], Muhammad Younas[2*], Shaista Shahzada[1], Muhammad Nadeem[2], Tahir Ali[2], Muhammad Javed Akhtar[2], Simone Pollastri[3], Ubaid-Ur- Rehman[2], Ibraheem Yousef[4] and Rao Tahir Ali Khan[2]

[1]Department of Physics Faculty of Basic and Applied Sciences, International Islamic University, Islamabad, Pakistan
[2]Physics Division, PINSTECH, P.O. Nilore, Islamabad 45650, Pakistan
[3]CERIC-ERIC, S.S. 14, Km 163.5 in Area Science Park, Basovizza 34149, Italy
[4]ALBA Synchrotron Light Source, Carrer de la Llum, 2-26, 08290 Barcelona, Spain

*Corresponding author: chuhan.pieas@gmail.com

Supporting Figures

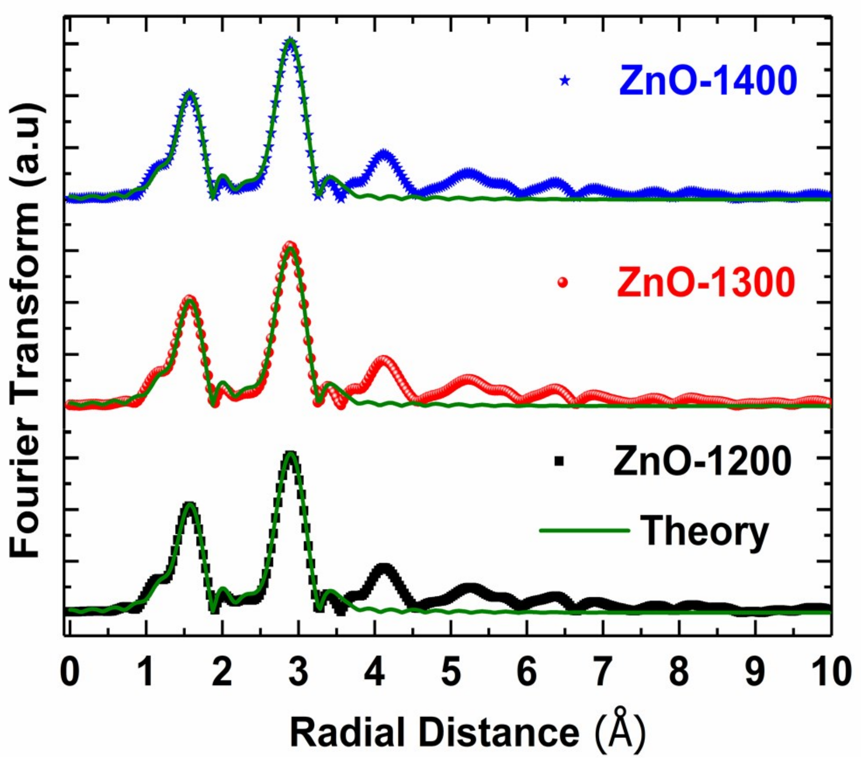

Fig. S1 Magnitude of the Fourier transform of the Zn K-edge spectra of ZnO sample sintered at different temperatures compared with theoretical model (solid lines).

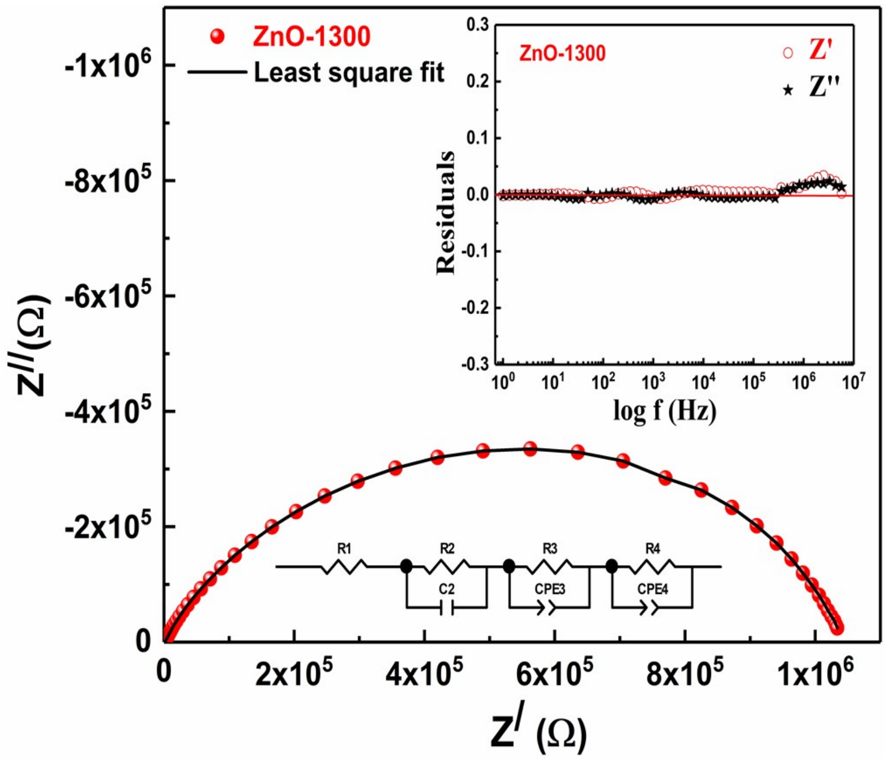

Fig. S2 Impedance plane plots (Z″ vs. Z′) of ZnO-1300 ceramic. The symbols and solid lines represent the data points and resultant fitted lines, respectively. The equivalent fitted model circuit is presented in lower insert. Upper right insert shows residuals between experimental and fitted data for the same sample.

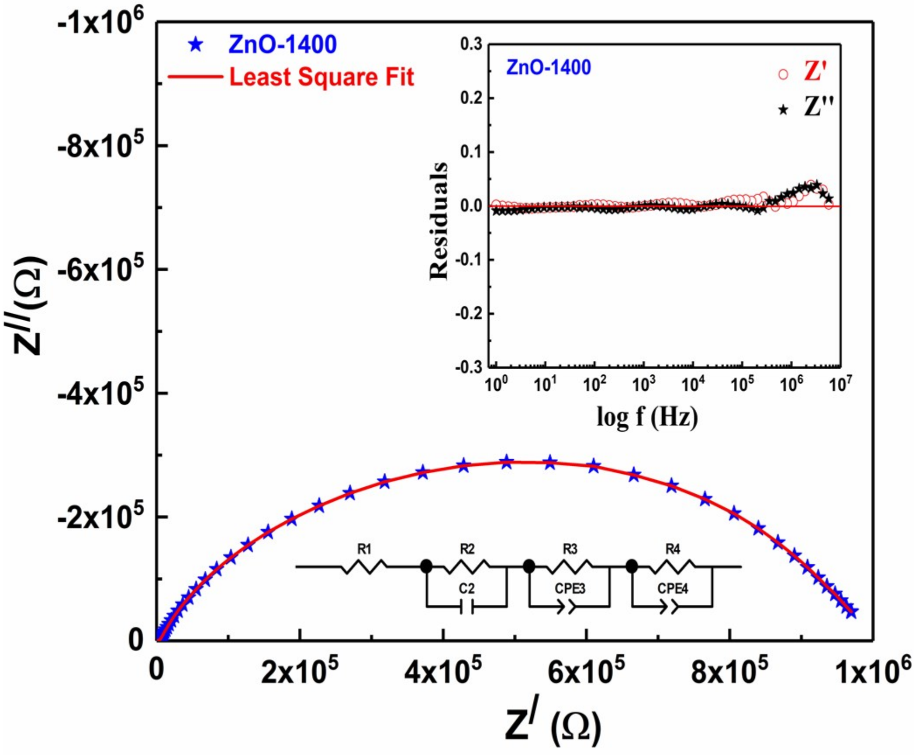

Fig. S3 Impedance plane plots (Z″ vs. Z′) of ZnO-1400 ceramic. The symbols and solid lines represent the data points and resultant fitted lines, respectively. The equivalent fitted model circuit is presented in lower insert. Upper right insert shows residuals between experimental and fitted data for the same sample.

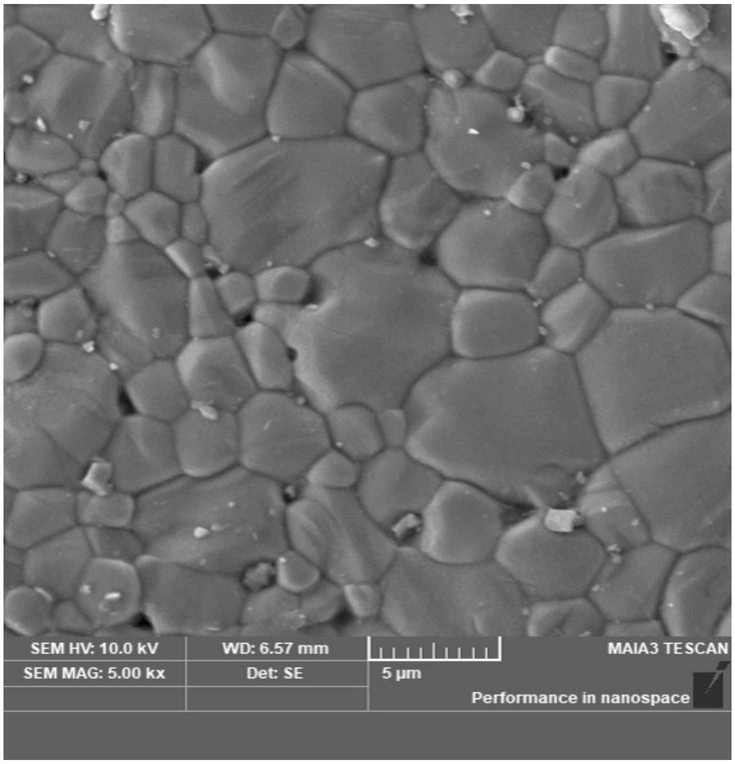

Fig. S4 High magnification SEM micrograph of ZnO-1200 ceramic.

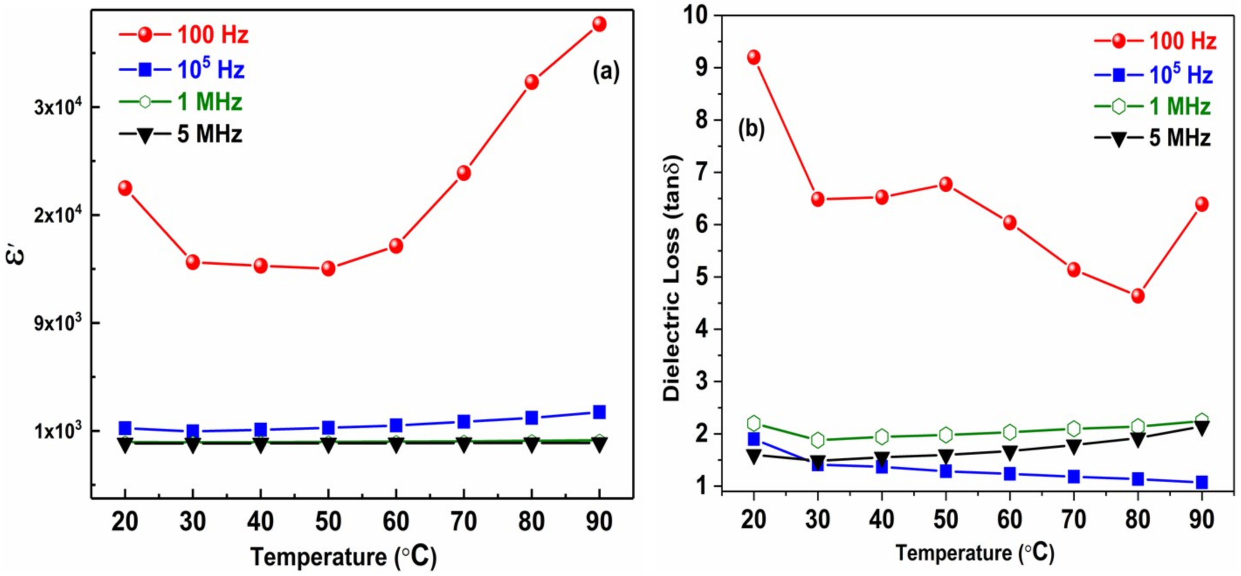

Fig. S5 (a) dielectric constant (ε′) vs. temperature and (b) dielectric loss (tanδ) vs. temperature at selected frequencies for ZnO-1200 ceramic.

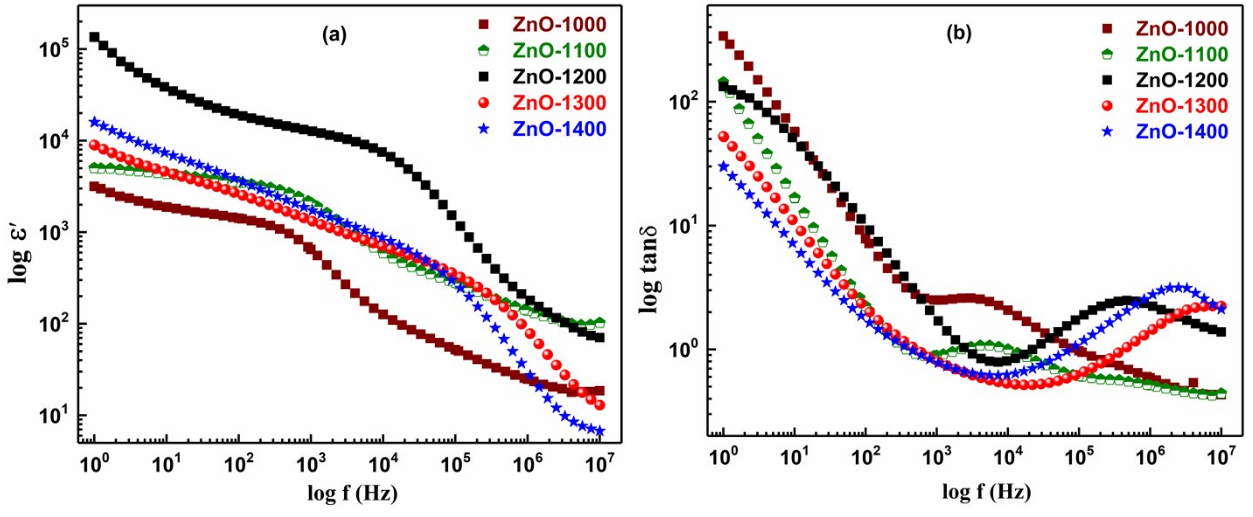

Fig. S6 Bod plots for ZnO sample sintered at 1000ºC-1400ºC in (a) log ε′ vs. log(f) and (b) log tanδ vs. log(f)) formalisms.
